# Echocardiographic reference ranges of myocardial work indices from the HUNT4Echo study

**DOI:** 10.1093/ehjimp/qyaf159

**Published:** 2025-12-24

**Authors:** Ingrid Yttervoll, Andreas Østvik, John Nyberg, Idar Kirkeby-Garstad, Even Olav Jakobsen, Petter Aadahl, Bjørnar Grenne, Håvard Dalen

**Affiliations:** Department of Circulation and Medical Imaging, Norwegian University of Science and Technology, Mailbox 8905, Trondheim 7491, Norway; Department of Circulation and Medical Imaging, Norwegian University of Science and Technology, Mailbox 8905, Trondheim 7491, Norway; Department of Cardiology, St. Olavs University Hospital, Trondheim, Norway; Medical Image Analysis, Health Research, SINTEF Digital, Trondheim, Norway; Department of Circulation and Medical Imaging, Norwegian University of Science and Technology, Mailbox 8905, Trondheim 7491, Norway; Department of Circulation and Medical Imaging, Norwegian University of Science and Technology, Mailbox 8905, Trondheim 7491, Norway; Department of Circulation and Medical Imaging, Norwegian University of Science and Technology, Mailbox 8905, Trondheim 7491, Norway; Department of Cardiology, St. Olavs University Hospital, Trondheim, Norway; Department of Circulation and Medical Imaging, Norwegian University of Science and Technology, Mailbox 8905, Trondheim 7491, Norway; Department of Circulation and Medical Imaging, Norwegian University of Science and Technology, Mailbox 8905, Trondheim 7491, Norway; Department of Cardiology, St. Olavs University Hospital, Trondheim, Norway; Department of Circulation and Medical Imaging, Norwegian University of Science and Technology, Mailbox 8905, Trondheim 7491, Norway; Department of Cardiology, St. Olavs University Hospital, Trondheim, Norway; Department of Medicine, Nord-Trøndelag Hospital Trust, Levanger, Norway

**Keywords:** pressure–strain loop, left ventricular mechanics, noninvasive hemodynamics, population-based cohort

## Abstract

**Background:**

Reference ranges for myocardial work indices are limited by the scarcity of data from the clinically relevant group of elderly individuals. Myocardial work indices constitute load-adjusted left ventricular function, and main components include global work index (GWI), global constructive work (GCW), global wasted work (GWW), and global work efficiency (GWE).

**Aims:**

To establish reference values for myocardial work indices and pressure-strain loop shape from guideline-directed recordings in a healthy population spanning a broad age range.

**Methods and results:**

We assessed myocardial work in healthy participants from the HUNT4Echo study. Global longitudinal strain was obtained by two expert cardiologists using two-dimensional speckle tracking, and systolic blood pressure from brachial measurements. Timing of valve events was performed by a single observer supervised by the expert cardiologists. Among 1239 participants (mean age 57, 55% female), reference ranges for myocardial work indices were as follows: GWI 1367–2583 mmHg%, GCW 1664–2972 mmHg%, GWW 38–328 mmHg%, and GWE 88–98%. Age was associated with lower GWI and GWE, and higher GCW and GWW (all *P* < 0.05). Sex influenced myocardial work indices, with somewhat higher GWI and GCW in females (*P* ≤ 0.001). The shape of the pressure-strain loops was narrower in older groups, while GWI (the area encompassed by the loop) remained constant across age groups.

**Conclusion:**

Myocardial work indices were influenced by age and sex, but effects were minor and have limited clinical relevance. Despite preserved GWI by higher age, the pressure-strain loop shape changes significantly – underscoring the importance of integrating strain and afterload when assessing left ventricular function.

**Trial registration number:**

Not applicable

## Graphical Abstract

**Graphical Abstract:**
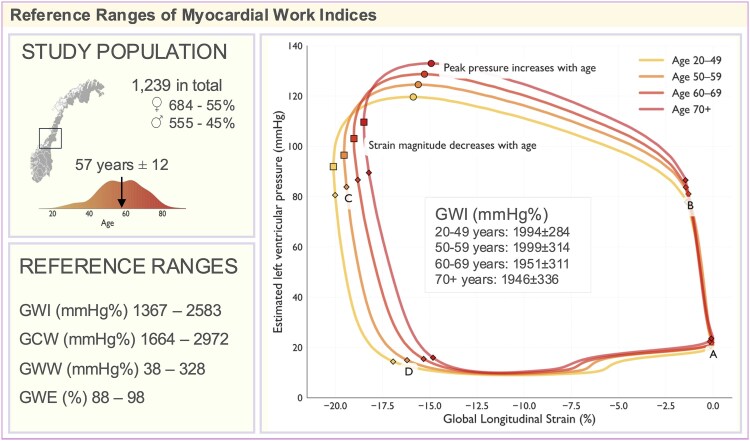
Reference Ranges of Myocardial Work Indices. Reference ranges for myocardial work indices are defined as the 95% reference interval calculated as mean ± 1.96 SD for global work index (GWI) and global constructive work (GCW) or the 2.5th and 97.5th percentiles for global wasted work (GWW) and global work efficiency (GWE). Pressure-strain loops are presented as the average in age groups. The average global work index (area of the loop) did not differ between age groups (*P* = 0.059), but the loop shapes did (all *P* < 0.031). Maximal systolic pressure (circle) and absolute global longitudinal strain (square) are marked. Time points indicated at mitral valve closure (A), aortic valve opening (B), aortic valve closure (C), and mitral valve opening (D).

## Introduction

A comprehensive, non-invasive evaluation of left ventricular systolic function remains a fundamental goal in cardiovascular medicine. The gold standard for evaluating left ventricular systolic function is analysis of pressure-volume loops.^1^ The area encompassed by the pressure-volume loops represents stroke work^2^—the energy transferred to the aorta during systole.^3^ However, despite their value, pressure-volume loops are not routinely used in clinical practice due to the need for simultaneous measurement of left ventricular volume and pressure, which are both invasive and cumbersome to obtain. Lately, Russel *et al.*^4^ developed a method for non-invasive assessment of left ventricular function by analysis of pressure-*strain* loops. Pressure-strain loops represent the relationship between estimated left ventricular pressure and myocardial deformation.

The pressure component of pressure-strain loops is derived from non-invasive brachial systolic blood pressure measurements extrapolated via cardiac valve event timing to create a continuous pressure curve. Pressure-strain loops have demonstrated excellent correlation with invasively measured intraventricular pressure curves.^4^ The strain component represents myocardial deformation from volume changes within the cardiac chambers.

The area encompassed by the pressure-strain loop is defined as the global work index (GWI), which correlates strongly with myocardial glucose metabolism.^4^ GWI is one of four established myocardial work indices,^5^ alongside global constructive work (GCW), global wasted work (GWW), and global work efficiency (GWE). Anticipating a relatively constant afterload and defining cardiac cycle phases by valvular events, GCW represents physiologically effective work (lengthening during diastole and shortening during systole). Conversely, GWW represents ineffective work (shortening during diastole and lengthening during systole). GWE is simply the ratio of GCW to the sum of GCW + GWW.

While several studies have established normative values for myocardial work indices,^5,6^ previous attempts have been small or dominated by young participants, which does not reflect the majority of patients encountered in clinical cardiology. Therefore, our objective was to establish comprehensive reference values for myocardial work indices using data from a large, prospective population health study and to study the effects of age and sex on these indices to enhance their clinical value.

## Methods

### Study population

The study population was derived from the HUNT4Echo study, a substudy of the larger Norwegian Nord-Trøndelag Health Study (HUNT). HUNT4 represents the fourth wave of the longitudinal HUNT population study, with data collection for the HUNT4Echo study occurring between October 2017 and June 2018.

Clinical measurements, including height, weight, blood pressure, and blood tests, were obtained on the screening day for the HUNT4 study. Blood pressure was measured with participants seated with their arms rested. Three measurements were obtained using the Dinamap Carescape V100 device (GE Healthcare) at intervals after 5 min rest, and the average of the final two readings was used as the representative value. The median (IQR) time delay between blood pressure measurements and the echocardiographic examination was 42 (32–59) days. Additionally, blood pressure was measured in the supine position twice during echocardiography in a random subsample of approximately one third. The averaged value is used in the analyses. Heart rate was measured as part of the echocardiographic examination. Body mass index was calculated by dividing weight (in kilograms) by the square of height (in meters). Information on smoking status and use of medications for hyperlipidaemia, asthma/chronic obstructive pulmonary disease, anxiety, depression, and allergies was collected via self-administered questionnaires. To ensure that all analysed participants were healthy, we excluded individuals with prior cardiac or metabolic conditions (e.g. atrial fibrillation, diabetes mellitus), those using antihypertensive medication, and those with incidental echocardiographic abnormalities. Additionally, participants with systolic blood pressure (SBP) > 160 mmHg at the study visit were excluded. In the supplementary myocardial work indices and factors analyses, we also excluded participants with SBP >140 mmHg. The procedures for acquiring demographic data have been previously described in detail.^7^

### Echocardiography acquisitions

The echocardiographic examinations were conducted according to the European Association of Cardiovascular Imaging (EACVI) and American Society of Echocardiography (ASE) guidelines.^8^ All participants were positioned in the left lateral decubitus position. The examinations were performed using Vivid E95 scanners (GE HealthCare) using 4V-D and M5S-D phased-array transducers. All examinations were conducted by two experienced sonographers (>2000 examinations each) with guideline-directed focused recordings for each of the four chambers. The examinations took place at Levanger Hospital (Levanger, Norway). Left ventricular global longitudinal strain (GLS) was analysed in three different left ventricular-focused apical views (four-chamber, two-chamber, and long-axis) with the use of the two-dimensional strain speckle tracking application by two expert cardiologists (BG and HD), both EACVI-certified in transthoracic echocardiography, >10 000 examinations, >15 years of experience in strain imaging. Comprehensive details are previously described.^9^

### Myocardial work analysis

All myocardial work analyses were performed on EchoPAC (version 206, GE HealthCare) by a single observer (IY) under continuous supervision of the same two expert cardiologists. The sitting blood pressure obtained on screening day was used in the myocardial work analysis for all participants. Cardiac valve event timing was defined as follows: (i) Visual assessment was used if both mitral and aortic valve events (opening and closing) were clearly visible on the apical long-axis view. The mitral valve closure and aortic valve closure were defined as the first frame where the valve was closed, and subsequently, for the aortic valve opening and mitral valve opening as the first frame where the valve was open, as recommended.^8^ (ii) If the mitral or aortic valve events were not visible, we used, respectively, visual assessment in B-mode 4-chamber view or spectral Doppler imaging.^6^ Spectral Doppler quality, based on signal resolution and probe positioning, was evaluated, and the timing of events was measured using the signals with the highest quality. The aortic valve opening was annotated at the opening-click if visible or at the onset of flow through the left ventricular outflow tract. The aortic valve closure was annotated at the closing click if visible or at the end of flow. If needed, the timing of valve events was adjusted in apical long-axis B-mode images during myocardial work indices analysis, as recommended by the vendor.

To account for heart rate variations between spectral Doppler and apical long-axis B-mode, we annotated at least two cycles and ensured that the measurements differed by no more than 12 milliseconds. This kept the difference between cycles mostly within one frame, as the mean (SD) frame rate was 71(4) frames per second. The software also has specific heart rate variation limitations with a cutoff at 30% difference between acquisitions used for analysis.^10^ We excluded participants in whom the aortic valve was not visible on B-mode apical long-axis images, whose spectral Doppler images were of insufficient quality for analysis, or who had fewer than two Doppler cycles available for annotation. We also excluded participants whose mitral valve was not visible in either the apical long-axis or the four-chamber view.

### Ethical approval

The HUNT4Echo study was approved by the Mid-Norway Regional Committee for Medical and Health Research Ethics (13 083). The study was carried out according to the principles of the Declaration of Helsinki. The security and handling of personal data of the participants was approved by the institutional personal data officer at Levanger Hospital, St. Olavs Hospital, and the Norwegian University of Science and Technology (Trondheim, Norway).

### Feasibility and reproducibility

Feasibility was calculated as the number of successfully calculated myocardial work indices among all included healthy participants. Myocardial work indices were considered unsuccessful if echocardiographic valve event timing was impossible due to poor image quality, if no strain curves were approved, or if heart rate variation was too high.

Inter-observer analysis was performed by comparing the reader’s (IY) event timing and GWI with analyses done by the same two expert cardiologists in a subset of 87 participants (86 participants for HD due to event timing being faulty in one participant, *n* = 120 before applying exclusion criteria). The two experienced cardiologists performed both strain and event timing, while IY performed only event timing, allowing for evaluation of the variability of both event timing and myocardial work indices. Intra-observer analysis was conducted by comparing both event timing and myocardial work indices results in a subset of 30 participants by IY, with at least four weeks of separation. Both subsets were selected using a weighted random sampling approach, and we iteratively refined our selection until the distribution of the investigated metrics did not differ significantly from the total study population.

### Pressure-strain loop shape analysis

Pressure-strain loops were used to characterize age-related differences in myocardial work morphology. For each participant, the pressure-strain loop was time-normalized to a single cardiac cycle and resampled to 1000 points through linear interpolation, using piecewise segments of 250 samples between valve events. Pointwise mean loops were computed for each age group and, for the reproducibility analysis, for each observer in the inter- and intra-observer datasets. Confidence intervals (±SD) for the mean pressure-strain loops were calculated using the covariance of the GLS and pressure waveforms forming the loops.

Pairwise comparisons of loop shape were evaluated using dynamic time warping (DTW) and Procrustes alignment. DTW was applied with Euclidean distance and an unconstrained full cycle warping path, capturing both temporal and geometrical mismatch between loops. Procrustes alignment was performed using translation, rotation, and isotropic scaling to provide a scale-invariant measure of geometric shape disparity.

To facilitate reproducibility and enable reuse in future methodological developments, code used for pressure–strain loop preprocessing, alignment, and statistical comparison is available at: https://gist.github.com/androst/553a60cfb60232f3e14923940b17fc7f

### Statistical analysis

We used *P* < 0.05 as the significance level. Continuous, normally distributed variables are expressed as mean ± SD; non-normally distributed variables as median (IQR); and categorical variables as n (%). Normality was evaluated by visual inspection of QQ-plots and histograms. Equality of variance was evaluated using Levene’s test. Outliers were identified with Tukey’s method. Reference intervals are reported as mean ± 1.96 SD for normally distributed variables and 2.5th and 97.5th percentiles for skewed variables. When comparing two groups, we used a two-sample *t*-test and the Mann-Whitney U test as appropriate, and for multiple comparisons, we used one-way ANOVA with Tukey’s post-hoc test or Kruskal–Wallis with Bonferroni corrected Dunn’s post-hoc test as appropriate. Proportions were analysed using the chi-square test. To assess the effect of age, we applied univariable linear regression models. To test whether age retained as an independent association with myocardial work indices, we applied multivariable linear regression and assessed the independent association for SBP, GLS, cardiac valve event timing, sex, body mass index, and heart rate on myocardial work indices. Specifically for cardiac valve event timing analysis, we excluded participants where the mitral valve closure and aortic valve openings were not in the correct sequence.

Before finalizing model selection, we compared linear models with more complex cubic spline regression models (four degrees of freedom) using ANOVA. For the multivariable analyses, the cubic spline models did not demonstrate superior fit (all *P* > 0.063). For the univariable analyses, the spline models were significantly better for GWW and GWE; however, improvements in R² were minimal (from 0.061 to 0.071 and from 0.053 to 0.062, respectively). Therefore, linear regression models were ultimately used for all analyses.

We also performed a multivariable analysis that included an age × sex interaction term. Regression models were used to assess the effect of a short time delay (≤31 days between blood pressure measurements and the echocardiographic examination) on myocardial work indices and to determine whether time delay modified the effects of sex and age.

We validated the assumptions of the linear regression model by first confirming linear relationships through visual inspection of residuals against fitted values. Independence of observations was ensured by the study design. Homoscedasticity was tested using the Breusch–Pagan test; if heteroscedasticity was present, robust standard errors were used to calculate test statistics. We assessed multicollinearity using variance inflation factor analysis, with values <5 considered acceptable. To achieve normality of residuals, GWW was log-transformed, and GWE was logit-transformed; however, all values in the plots are shown on the original scale.

Significance of pressure-strain loop shape differences was assessed by permutation testing (10 000 permutations), in which age-group or observer labels were randomly reassigned while preserving group sizes. *P*-values were computed as the proportion of permuted DTW or Procrustes distances greater than or equal to the observed distance. For age-group comparisons, we report the larger of the DTW- and Procrustes-based *P*-values; for the reproducibility analysis, we report the smaller *P*-value.

Inter- and intra-observer agreement was evaluated using Bland-Altman statistics, as well intra-class correlation (ICC) for single random raters. All analyses were performed using Python 3.9.22 with the following libraries: NumPy 2.0.2 and pandas 2.2.2 for data manipulation; tlearn 0.6.3, SciPy 1.13.1, statsmodels 0.14.4, and pingouin 0.5.5 for statistical analyses and Matplotlib 3.9.4 and seaborn 0.13.2 for visualizations.

## Results

**Figure 1:**
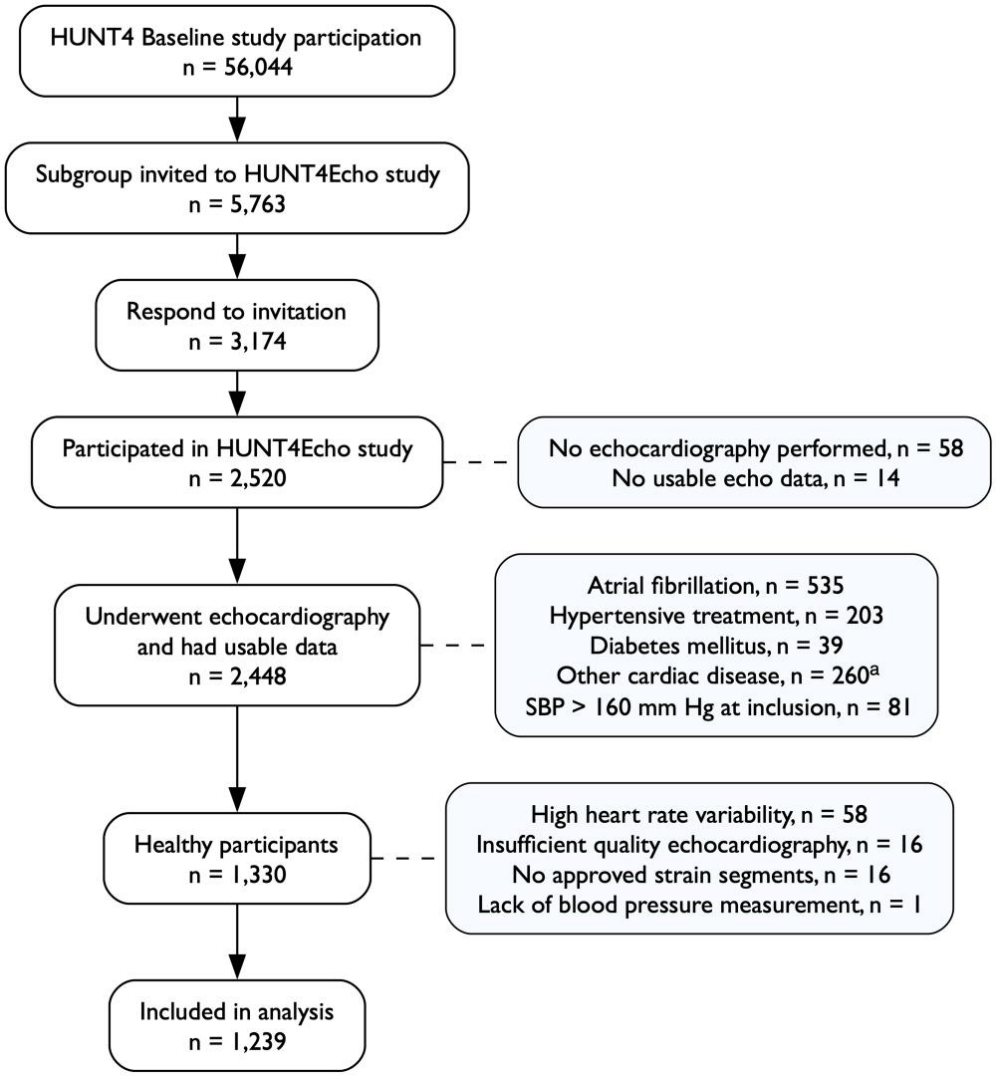
Flowchart of the study. ^a^From participants’ self-reports, validation of medical record files, or echocardiographic analyses. Abbreviations: HUNT4Echo, echocardiographic substudy of the 4th wave of the Trøndelag Health Study; n, numbers; SBP, systolic blood pressure.

The study consisted of 1239 participants (684 (55%) females) with age of 57 ± 12 years. We excluded 91 (7%) participants for reasons displayed in *Figure 1*. Excessive heart rate variation accounted for 58 (64%) of the excluded participants, with most 47 (81%) due to software-specific limitations and the remainder 11 (19%) due to beat-to-beat variation in the spectral Doppler signals. Visual assessment was the predominant technique used for valve-event timing 1104 (89%). The baseline characteristics of the study population are displayed in *Table 1*. Use of regular medications was low (<11%), and included drugs for hyperlipidaemia, asthma, chronic obstructive pulmonary disease, anxiety, depression, and allergies.

**Table 1:** Baseline characteristics of the study population

Characteristics	Overall, *n* = 1239	Female, *n* = 684	Male, *n* = 555	P (sex)
Age, years	57 ± 12	57 ± 12	57 ± 12	0.99
Height, cm	172 ± 9	166 ± 6	180 ± 7	<0.001
Weight, kg	76 (19)	67 (13)	84 (13)	<0.001
Body mass index, kg/m²	25.2 (4.5)	24.5 (4.8)	25.8 (3.5)	<0.001
Heart rate, beats/min	66 (15)	68 (14)	64 (14)	<0.001
SBP, mmHg	124 (20)	122 (20)	127 (19)	<0.001
GLS, %	−19.5 ± 2.3	−19.9 ± 2.3	−19.0 ± 2.2	<0.001
GWI, mmHg%	1975 ± 310	2024 ± 301	1915 ± 311	<0.001
GCW, mmHg%	2318 ± 334	2345 ± 332	2284 ± 333	0.001
GWW, mmHg%	104 (73–146)	106 (73–147)	103 (74–143)	0.641
GWE, %	95 (94–96)	95 (94–96)	95 (94–96)	0.693
LVEF, %	60.6 (6.1)	61.0 (6.3)	60.1 (6.3)	0.008
MVC, ms	25 (13)	24 (12)	25 (15)	0.313
AVO, ms	80 (18)	80 (18)	81 (18)	0.487
AVC, ms	369 (31)	374 (30)	361 (29)	<0.001
MVO, ms	465 (35)	467 (36)	462 (34)	0.010
HbA₁c, mmol/mol	33 (4)	33 (4)	34 (3)	<0.001
Cholesterol, mmol/L	5.64 ± 1.03	5.68 ± 1.08	5.58 ± 0.97	0.081
Current smoker, (%)	37 (3.0)	27 (3.9)	10 (1.8)	0.043

Values are presented as mean ± SD, median (IQR), or n (%). Abbreviations: AVC, aortic valve closure; AVO, aortic valve opening; GCW, global constructive work; GLS, global longitudinal strain; GWE, global work efficiency; GWI, global work index; GWW, global wasted work; HbA₁c, glycosylated hemoglobin; MVC, mitral valve closure; MVO, mitral valve opening; LVEF, left ventricular ejection fraction; n/*n*, number; SBP, systolic blood pressure.

Overall, systolic blood pressure was 124 ± 13 mmHg in the sitting position and 131 ± 16 mmHg in the supine position (*n* = 423). In a secondary analysis restricted to participants with SBP <140 mmHg, all myocardial work indices except for GWE were significantly lower compared to all included participants (all *P* > 0.001, for GWE *P* = 0.077, Supplementary data online, *Table S1*). There was no statistically significant association between myocardial work indices and the time delay between the sitting blood pressure measurement and the echocardiographic examination (lowest *P*-value 0.280). Myocardial work indices did not differ significantly between the short time delay group (≤ 31 days, *n* = 301) and the rest (all *P* > 0.064). Outliers had higher median SBP (139 mmHg vs. 124 mmHg) and lower absolute median GLS (−18.9% vs. −19.6%) compared with non-outliers, and dispersion was somewhat higher in older age groups (see Supplementary data online, *Table S2*).

### Feasibility, reproducibility and repeatability

**Figure 2:**
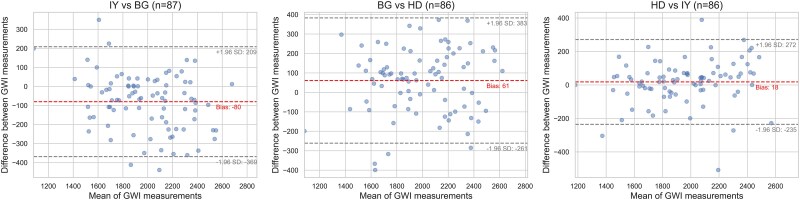
Agreement of GWI measurements between observers. The solid points represent paired observer comparisons, and dashed lines indicate the mean bias and limits of agreement (±1.96 SD). IY, BG, and HD were observers. Abbreviations: GWI, global work index.

Myocardial work indices were feasible in 1239 (93%) of 1330 participants. The lowest ICC among all myocardial work indices was 0.73 (95% CI: 0.62–0.81), and no bias exceeded 80 mmHg%, with limits of agreement ranging from −209 to 369 (±1.96 SD) (*Figure 2*). In addition, when controlling for the different modes used for event timing (visual assessment or Doppler) the lowest ICC was 0.77 (95% CI: 0.77–0.89). As recently published,^9^ the ICC for left ventricular GLS was 0.84 (95% CI: 0.77–0.89). Complete intraobserver and interobserver agreement results are provided in Supplementary data online, *Tables S3* and *S4*, respectively. Supplementary data online, *Table S5* shows valve event timing variability before and after multimodality control.

### Pressure-strain loops

**Figure 3:**
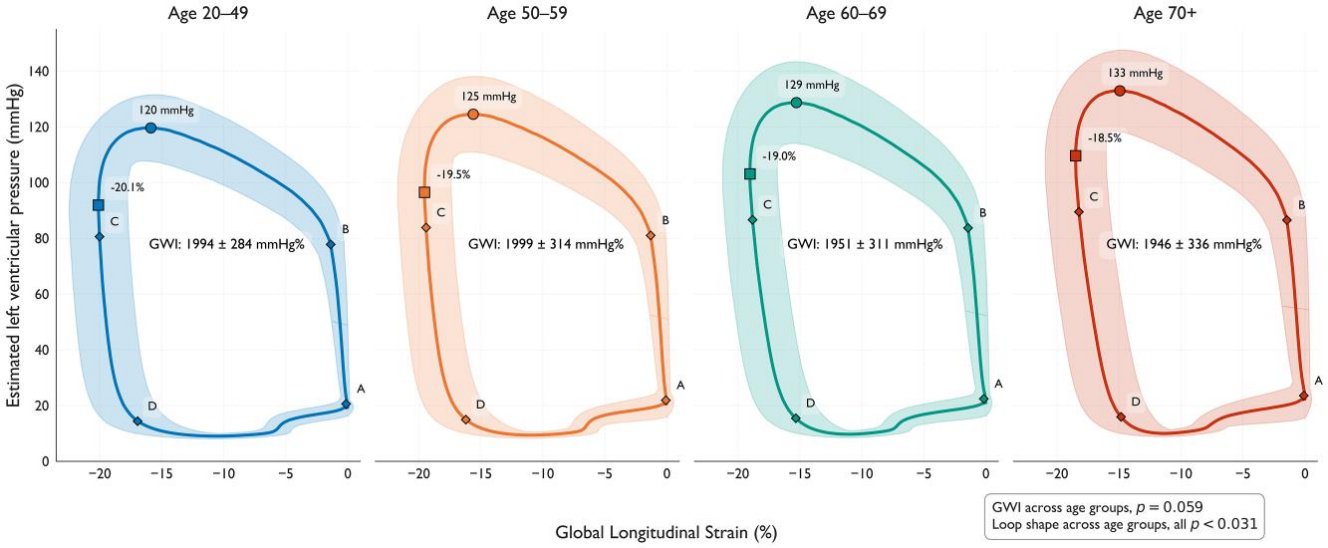
Pressure-strain-loop analysis by age group. Thin coloured lines represent the average pressure-strain relationship for each age group, with surrounding shaded areas indicating +/− 1SD. Boxes mark peak global longitudinal strain, and circles peak systolic blood pressure. Time points annotated at mitral valve closure (*A*), aortic valve opening (*B*), aortic valve closure (*C*), and mitral valve opening (*D*). Abbreviations: GWI, global work index.

The averaged pressure-strain loop shapes differed significantly across age groups, all *P*-values <0.001 except for the 60–69 years vs. 70+ years groups, *P* = 0.031. By visual inspection, the loop shape narrowed, related to lower peak GLS, and stretched, related to higher SBP with higher age (*Graphical Abstract* and *Figure 3*). However, GWI, which represents the area of the pressure-strain loops, showed a small but significant effect of age. However, this age effect was not significant when assessed according to categorical age groups (*P* = 0.059). The factors comprising pressure-strain loops differed significantly (all *P* < 0.001), absolute GLS was lower, and SBP was higher in older age groups. The average pressure-strain loop shapes showed no significant intra-observer or inter-observer differences (*P* > 0.16; Supplementary data online, *Figures S1* and *S2*).

### Myocardial work indices related to age

With higher age, we observed significant differences in all myocardial work indices. Higher age was associated with lower GWI (β −1.45) and GWE (logit-transformed β −0.009), and higher GCW (β 2.32) and GWW (log-transformed β 0.011). After back-transformation, one year of higher age corresponded to a relative 1.1% higher GWW and 0.9% lower odds of maintaining GWE. In the fully adjusted model, the associations between myocardial work indices and age were preserved for all myocardial work indices except GWW (*P* = 0.114). The β-coefficients with corresponding *P*-values from the multivariable analysis are presented in Supplementary data online, *Table S6*.

**Figure 4:**
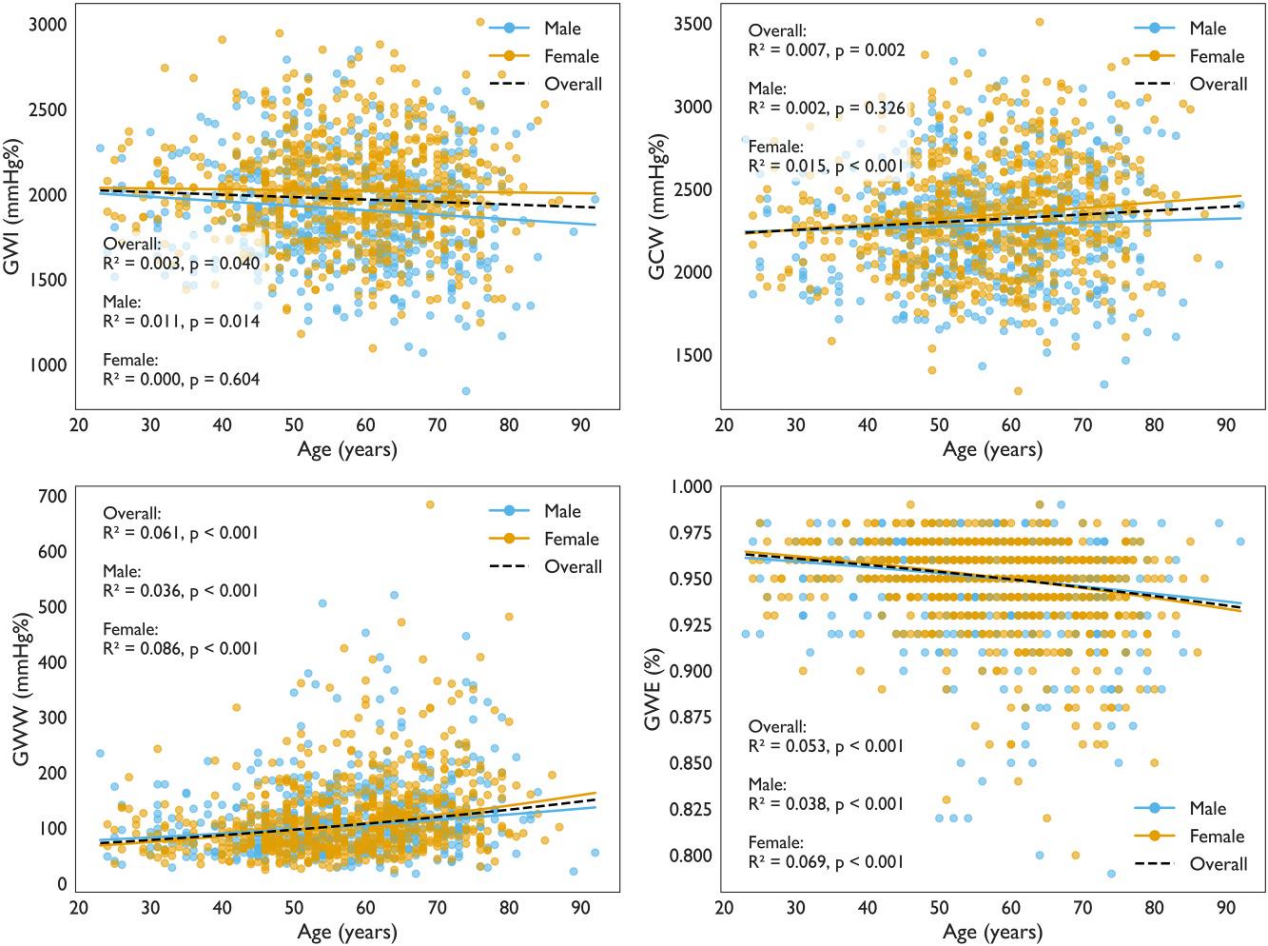
Myocardial work indices related to sex and age. Scatter plots showing the relationship between age (*x*-axis) and myocardial work indices (*y*-axis) with linear regression lines for the overall population (black dotted line), females (orange line), and males (blue line). *R*² values and *P*-values are displayed for each group. Abbreviations: GCW, global constructive work; GWE, global work efficiency; GWI, global work index; GWW, global wasted work.

However, while age significantly affected GWI overall, this relation was not significant in female participants when sexes were analysed separately (*P* = 0.604). Similarly, GCW was not significantly associated with age in male participants (*P* = 0.326). Furthermore, there was no significant age × sex interaction (all *P* > 0.105). The univariable linear regression analyses of myocardial work indices by age and gender are presented in *Figure 4*. Short time delay between blood pressure and echocardiography did not significantly affect the age effect (all *P* > 0.165, Supplementary data online, *Table S7*).

### Myocardial work indices related to sex

We observed significant differences in GWI and GCW between males and females, with females having a higher average GWI (2024 ± 301 mmHg% vs. 1915 ± 311 mmHg%) and GCW (2345 ± 332 mmHg% vs. 2284 ± 333 mmHg%) than males, both *P* ≤ 0.001. For GWI, significant differences were also observed in age-group sub-analyses (all *P* ≤ 0.032). For GCW, significant differences were only observed in the 60–69 years group (*P* = 0.011). We found no significant sex differences in either GWW or GWE, neither overall nor within any age-group sub-analyses (all *P* ≥ 0.309).

Short time delay between blood pressure and echocardiography did not significantly affect the sex effect on GWI and GCW (all *P* > 0.282, Supplementary data online, *Table S7*). After back-transformation, short time delay × sex was associated with a 14% decrease in GWW (95% CI: −25% to −3%, *P* = 0.019, Supplementary data online, *Table S7*). For GWE, the same interaction was associated with a 12% increase in the odds (95% CI: 0.21% to 26%, *P* = 0.046, Supplementary data online, *Table S7*).

**Figure 5:**
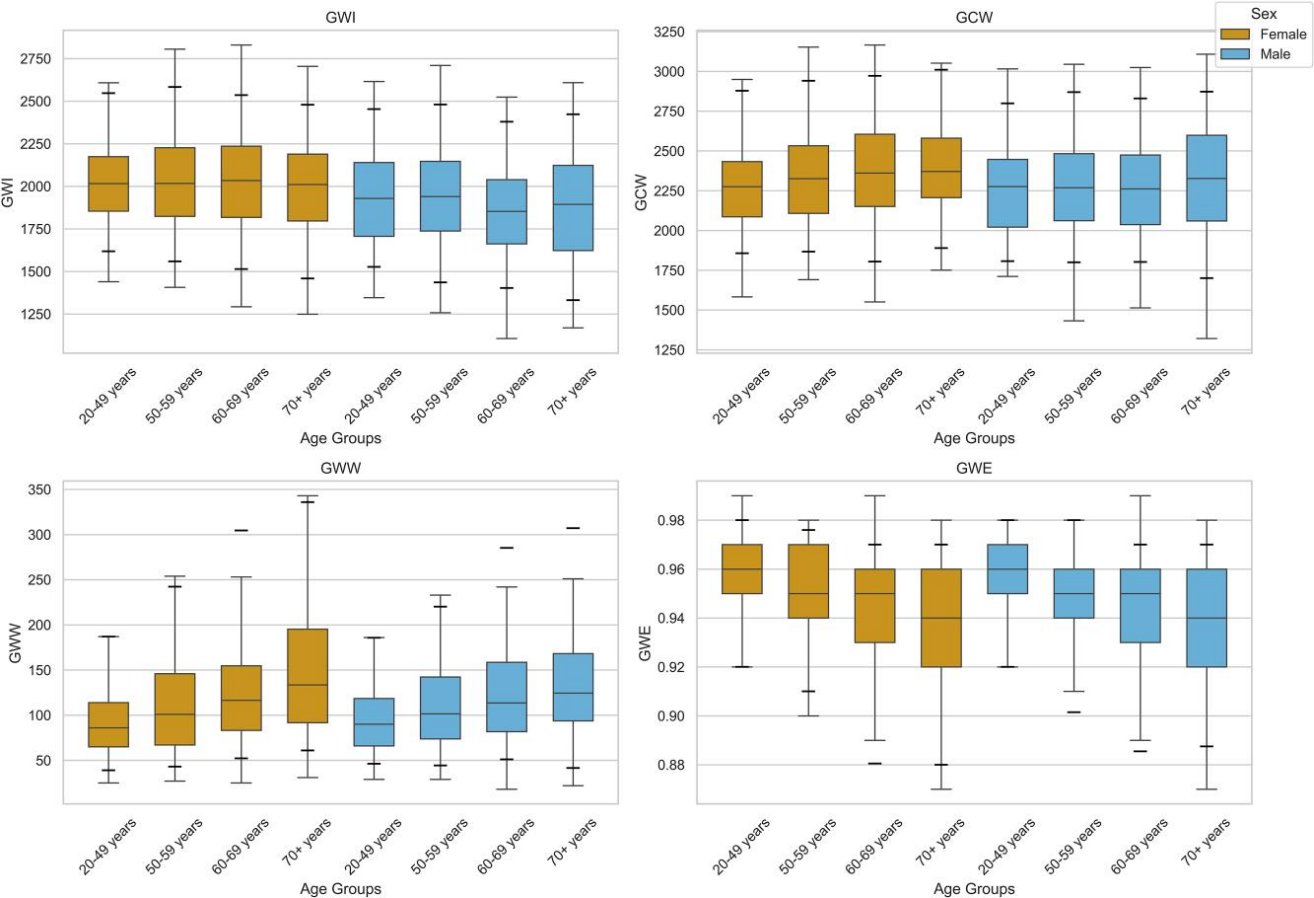
Percentile reference intervals for myocardial work indices. The central line in the box represents the 50th percentile (median), box edges represent the 25th and 75th percentiles. Whiskers extend to the final datapoint within 1.5 interquartile range. Additional lines represent the 5th and 95th percentiles. Abbreviations: GCW, global constructive work; GWE, global work efficiency; GWI, global work index; GWW, global wasted work.

All myocardial work indices stratified by age and sex are shown in *Table 2*, and the corresponding reference interval percentiles are displayed in *Figure 5*. Supplementary data online, *Table S8* compares our findings with principal studies in the field.

**Table 2:** Myocardial work indices and their factors by age and sex

Group	Age, years	*n*	GWI (mmHg%)	GCW (mmHg%)	GWW (mmHg%)	GWE (%)	SBP (mmHg)	GLS (%)
**Overall**	20–49	324	1994 ± 284^b^	2278 ± 308	88 (65–116)	96 (95–97)	119 (111–127)^a^	−20.2 ± 2.4^a^
	50–59	353	1999 ± 314^b^	2321 ± 332	101 (71–144)	95 (94–96)	124 (115–133)^a^	−19.7 ± 2.1^a^
	60–69	354	1951 ± 311^a^	2332 ± 344^b^	115 (83–157)	95 (93–96)	127 (119–139)	−19.2 ± 2.2^a^
	70+	208	1946 ± 336^b^	2352 ± 355	127 (93–185)	94 (92–96)	132 (123–144)	−18.7 ± 2.4
**Females**	20–49	181	2024 ± 278	2290 ± 306	86 (65–114)	96 (95–97)	115 (109–123)	−20.7 ± 2.4
	50–59	189	2040 ± 304	2343 ± 328	101 (67–146)	95 (94–97)	121 (112–129)	−20.2 ± 2.0
	60–69	202	2020 ± 314	2372 ± 359	117 (83–155)	95 (93–96)	125 (118–138)	−19.6 ± 2.3
	70+	112	2000 ± 307	2391 ± 321	134 (92–195)	94 (92–96)	132 (122–144)	−18.9 ± 2.4
**Male**	20–49	143	1956 ± 288	2263 ± 310	90 (66–119)	96 (95–97)	122 (115–131)	−19.6 ± 2.2
	50–59	164	1952 ± 319	2295 ± 335	102 (74–142)	95 (94–96)	128 (119–135)	−19.2 ± 2.1
	60–69	152	1858 ± 283	2278 ± 315	114 (82–159)	95 (93–96)	128 (121–140)	−18.7 ± 2.0
	70+	96	1883 ± 358	2306 ± 387	125 (94–168)	94 (92–96)	133 (124–145)	−18.4 ± 2.5

Values are presented as mean ± SD or median (IQR). Differences between female and ^a^*P* < 0.001; ^b^*P* < 0.05. Abbreviations: GCW, global constructive work; GLS, global longitudinal strain; GWE, global work efficiency; GWI, global work index; GWW, global wasted work; n, numbers; SBP, systolic blood pressure.

## Discussion

We have calculated myocardial work indices from a large, healthy population, expanding reference ranges to higher ages than previously reported. The three main findings are: (1) although pressure–strain loop shape differs significantly between age groups, GWI remains preserved, underscoring the importance of assessing both loop shape and area when evaluating myocardial work; (2) all myocardial work indices were influenced by increasing age, with lower GWI and GWE and higher GCW and GWW; (3) stratified by sex, the association of lower GWI with higher age persisted only in males, and similarly, higher GCW with higher age only in females. Overall, females had somewhat higher GWI and GCW than males.

### Feasibility, reproducibility, and repeatability

Our study demonstrated good reproducibility and repeatability metrics. In general, both interobserver and intraobserver variability were comparable to previous results for both valvular event timing and myocardial work indices.^11–13^

### Pressure-strain loops

The morphological alterations in pressure-strain loops with advancing age may be attributed to reduced absolute GLS, reduced left ventricular and aortic elastance, and increased SBP in older individuals.^9,14–16^ The alterations parallel similar age-related changes of pressure-volume loops, as displayed by Chen *et al.*^14^ Chen *et al.* also reported that ventriculo-arterial coupling was preserved with increasing age, which may explain why GWI in our study was preserved in the older age groups.

For clinical use, myocardial work indices should be interpreted against the age- and sex-specific percentiles in *Figure 5* and Supplementary data online, *Table S2*, where values between P5 and P95 indicate the expected range. If an index is within this range but the pressure–strain loop pattern resembles that of an older age group (higher pressure and lower strain), this could suggest increased afterload or early left ventricular dysfunction and should prompt further considerations.

However, it is important to emphasize that while GWI measured at rest is maintained, the functional reserve capacity during physical stress may be compromised with advancing age. Thus, further research on changes in pressure-strain loops during stress is needed to enhance their clinical utility.

### Myocardial work indices and their relation to age and sex

The overall reference ranges for myocardial work indices in this study aligned with previous findings, except for GWW, which were notably higher than previously reported.^5,6^ This discrepancy can be due to differences in the modality employed for valve event timing and differences in study population demographics. First, visual assessment yields a higher GWW than pulsed wave Doppler and even higher than tissue Doppler.^13^ We used visual assessment in 89% of cases, which could partly explain the higher GWW presented. Second, this study has an older population compared to other studies, and as GWW has been shown to be higher with higher age,^17^ participants' age may also partly explain the presented finding. The well-aligned GWW data from our youngest age group (20–49 years) and demographically similar age groups from previous studies,^11,12,17^ support that demographic differences are a significant contributor to differences between studies.

We found an effect of age on all myocardial work indices. This effect can be explained by age-related changes in GLS, SBP, and cardiac valve event time intervals.^9,15,18^ Previous studies have been inconsistent regarding how age influences myocardial work indices. Comprehensive details across these and the present study are shown in Supplementary data online, *Table S8*. In short, the inconsistency can be due to differences in timing methods, sample size, and differences in age and sex balance. Overall, all other studies had a narrower age span compared to our study, potentially reducing the power to detect differences in myocardial work indices by age.

The higher GWI among females is supported by others,^11,17,19^ and the finding of higher GCW in females is supported by Galli *et al.*^11^ The higher GWI and GCW in females could be explained by sex-based differences in large-artery stiffness.^20,21^ Redfield *et al.*^21^ showed that stiffness increases with age and even more so in females. A stiffer aorta demands an increased workload from the myocardium, which could explain the observed higher GWI and GCW in females.

Some previous studies also found significant sex influence on GWW and GWE,^12,19^ which we did not observe. Both the latter studies had a lower mean age than our study, suggesting that our observations represent an extension of observed trends into an older cohort.

In the clinical context, the statistical significance of age and sex effects on GWI appears of limited practical relevance. Specifically, 10 years of higher age corresponded to 38 mmHg% lower GWI, meaning a relative difference of just 2%. This raises the question of whether age-specific reference ranges for GWI are needed. Similarly, females had 129 mmHg% higher GWI than males, corresponding to a difference of just 6%. As shown by the relatively minor influence of age, we expect that the finding of no significant differences across categorical age groups for GWI was due to lack of statistical power.

### Myocardial work blood pressure measurement

Blood pressure recorded at screening in the sitting position was lower than the measurements from the supine position during echocardiography (mean difference 7 mmHg). A similar difference between sitting and supine positions has been shown previously.^22,23^ Thus, baseline measurements from the sitting position were used in the analyses for the best alignment to clinical practice.

### Strengths and limitations

Study strengths relate to the conduction of a large-scale population study of healthy participants with higher age than previously described. The echocardiographic assessment was conducted by experts in the field and with optimized echocardiographic acquisitions and analyses, as acknowledged in previous studies.^7,9^

The study also has some limitations. It was a single-centre study with a predominantly Caucasian cohort, and the myocardial work analysis was performed using scanners and software from a single vendor. These factors may limit the generalizability of the reference ranges, and caution is advised when applying them to other populations, vendors, or software versions. External validation across diverse populations and imaging platforms is therefore warranted.

Furthermore, sitting blood pressure measurements were not part of the echocardiographic study protocol. Ideally, blood pressure should be obtained simultaneously with strain imaging, and preferably within the same heartbeats. However, long-term changes in blood pressure are small, averaging less than 1 mmHg per year.^15^ Time delay did not significantly influence myocardial work indices, and the short time delay impact on the associations with age and sex was minor. Additionally, in a large cohort like ours (*n* = 1239), random variation due to the day-to-day fluctuations in blood pressure is expected to regress toward the mean at the population level, thereby not systematically biasing the reported reference values.

We used an SBP exclusion threshold of >160 mmHg rather than a lower, guideline-aligned cut-off to avoid a supranormal sample unrepresentative of community-dwelling older adults. This choice preserves generalizability but may increase dispersion and the number of outliers. Finally, we employed a multimodal approach for determining valve event timing, relying primarily on visual assessment. While this method may introduce temporal delays compared to Doppler-based measurements, potentially increasing variability in GCW and GWW, it more closely reflects real-world clinical practice and may enhance the applicability of our findings in routine settings.

## Conclusions

The present study suggests reference ranges for myocardial work indices in a large, healthy population, expanding the age ranges of previous studies, which may guide clinical interpretation and future research. Myocardial work indices were influenced by age and sex, but the effects were minor and likely of limited clinical relevance. Despite age-related changes in pressure–strain loop shape, GWI remains preserved, highlighting the importance of assessing both loop shape and area for the best evaluation of left ventricular function.

## Supplementary Material

qyaf159_Supplementary_Data

## Data Availability

The dataset used in this study is available through application via the HUNT4 data portal: https://www.ntnu.edu/hunt/research.

## Lead author biography

Ingrid Yttervoll is a medical and research programme student at the Norwegian University of Science and Technology (NTNU) in Trondheim, Norway. Her research interests focus on cardiovascular physiology, particularly hemodynamics and multimodal assessment of left ventricular function. She has contributed to projects that integrate echocardiography, non-invasive continuous blood pressure monitoring, and artificial intelligence to enhance cardiac assessment. Yttervoll is passionate about bridging the gap between clinical medicine and research, aiming to advance the imaging-based understanding of cardiac function. She is currently involved in both clinical and experimental studies focused on non-invasive assessment of left ventricular energetics.
