# Supplementary material for: Echocardiographic reference ranges of myocardial work indices from the HUNT4Echo study
Source: Eur Heart J Imaging Methods Pract. 2025 Dec 24;4(1):qyaf159. doi: 10.1093/ehjimp/qyaf159 (PMC12813916; doi:10.1093/ehjimp/qyaf159)
Supplement: qyaf159_Supplementary_Data [file qyaf159_supplementary_data.docx]

**SUPPLEMENTARY DATA**

**Supplementary Figure 1.** Interobserver Reproducibility for Pressure-Strain loops.


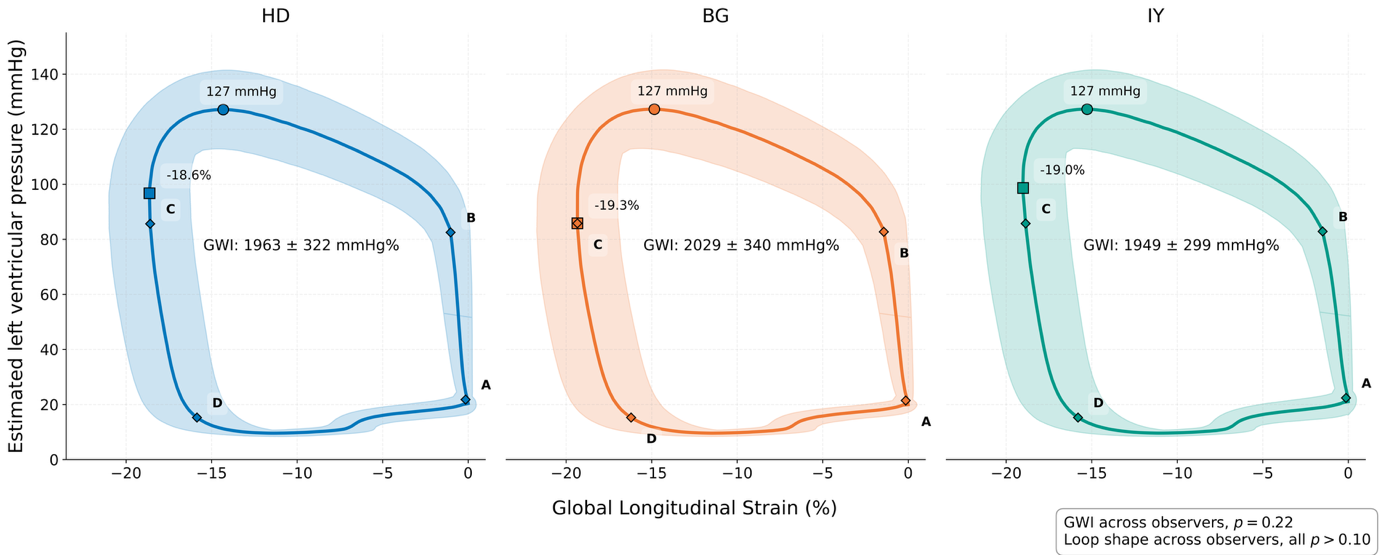


Thin coloured lines represent the average pressure-strain relationship for each observer (HD, BG or IY), with surrounding shaded areas indicating +/- 1SD. Boxes mark peak global longitudinal strain, and circles peak systolic blood pressure. Time points annotated at mitral valve closure (A), aortic valve opening (B), aortic valve closure (C) and mitral valve opening (D). Abbreviations: GWI, global work index. Abbreviations: BG, HD and IY, the three different observers; GWI, global work index.

**Supplementary Figure 2.** Intraobserver Repeatability for Pressure-Strain loops.

**
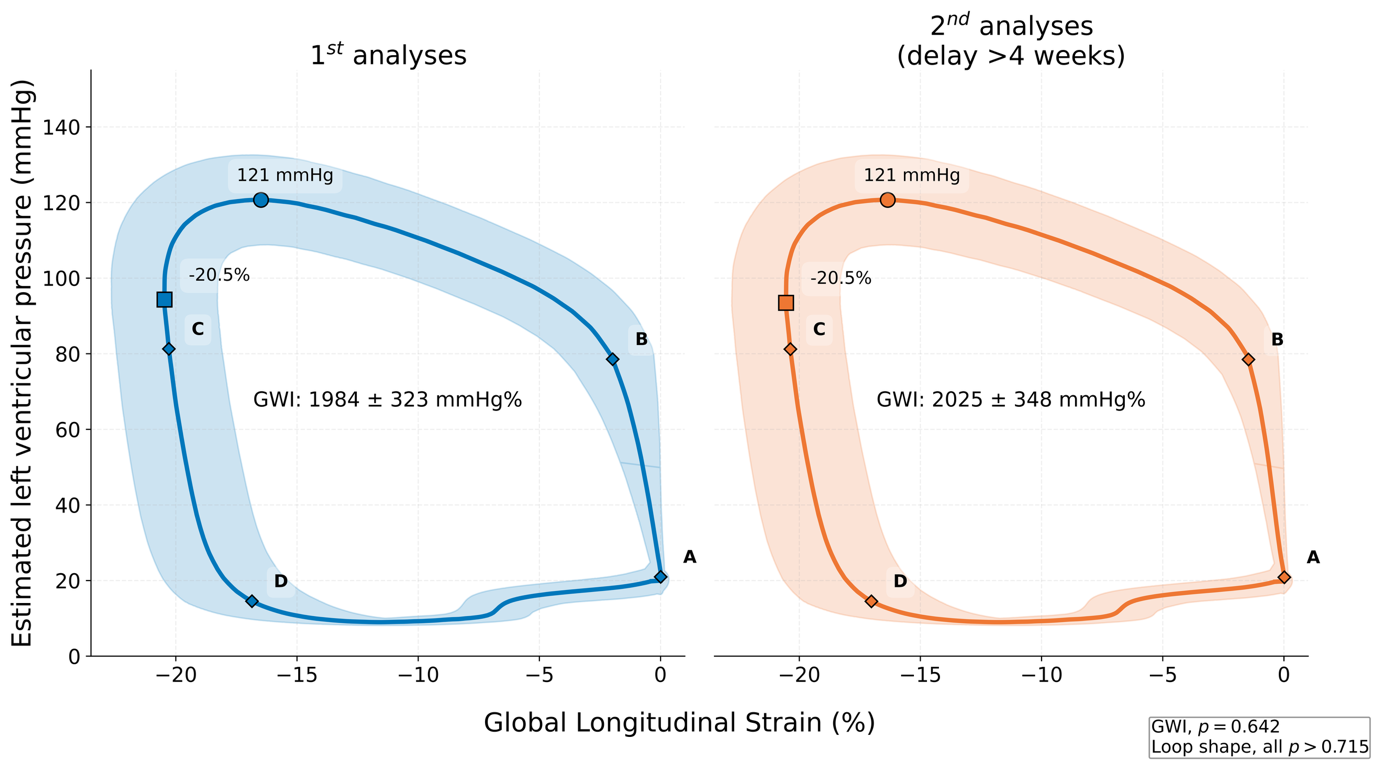
**

Thin coloured lines represent the average pressure-strain relationship for one observer (IY) at two different time points with surrounding shaded areas indicating +/- 1SD. Boxes mark peak global longitudinal strain, and circles peak systolic blood pressure. Time points annotated at mitral valve closure (A), aortic valve opening (B), aortic valve closure (C) and mitral valve opening (D). Abbreviations: GWI, global work index. Abbreviations: GWI, global work index.

**Supplementary Table 1.** Myocardial work indices and their factors by age and sex in participants with SBP <140 mmHg.

| **Group** | **Age, years** | **n** | **GWI (mmHg%)** | **GCW (mmHg%)** | **GWW (mmHg%)** | **GWE (%)** | **SBP (mmHg)** | **GLS (%)** |
| --- | --- | --- | --- | --- | --- | --- | --- | --- |
| **Overall** | 20-49 | 301 | 1968 ± 266^b^ | 2245 ± 283 | 84 (63-113) | 96 (95-97) | 118 (111-124)^a^ | -20.3 ± 2.3^a^ |
|  | 50-59 | 300 | 1946 ± 280^b^ | 2252 ± 284 | 96 (67-130) | 95 (94-97) | 121 (113-128)^a^ | -19.8 ± 2.1^a^ |
|  | 60-69 | 268 | 1878 ± 272^a^ | 2234 ± 297 | 108 (79-144) | 95 (93-96) | 123 (117-130)^b^ | -19.3 ± 2.1^b^ |
|  | 70+ | 132 | 1829 ± 313^b^ | 2214 ± 307 | 120 (89-172) | 94 (92-96) | 126 (118-131) | -18.9 ± 2.4 |
| **Females** | 20-49 | 171 | 2001 ± 258 | 2263 ± 281 | 85 (63-112) | 96 (95-97) | 114 (108-121) | -20.8 ± 2.3 |
|  | 50-59 | 165 | 1986 ± 272 | 2275 ± 280 | 99 (66-129) | 95 (94-97) | 118 (110-125) | -20.3 ± 2.0 |
|  | 60-69 | 155 | 1935 ± 264 | 2263 ± 303 | 112 (80-150) | 95 (93-96) | 122 (116-129) | -19.6 ± 2.2 |
|  | 70+ | 74 | 1886 ± 265 | 2262 ± 250 | 122 (87-163) | 94 (93-96) | 126 (119-131) | -19.1 ± 2.4 |
| **Male** | 20-49 | 130 | 1924 ± 272 | 2222 ± 285 | 84 (63-114) | 96 (95-97) | 121 (115-128) | -19.6 ± 2.2 |
|  | 50-59 | 135 | 1896 ± 282 | 2223 ± 288 | 95 (71-133) | 95 (94-96) | 124 (118-132) | -19.2 ± 2.1 |
|  | 60-69 | 113 | 1800 ± 265 | 2194 ± 284 | 102 (73-141) | 95 (94-96) | 125 (118-132) | -18.9 ± 1.9 |
|  | 70+ | 58 | 1756 ± 354 | 2154 ± 361 | 116 (93-188) | 94 (92-96) | 126 (118-131) | -18.5 ± 2.5 |

Values are presented as mean ± SD or median (IQR). ^a^ p < 0.001; ^b^ p < 0.05 for differences between females and males. Abbreviations: GCW, global constructive work; GLS, global longitudinal strain; GWE, global work efficiency; GWI, global work index; GWW, global wasted work; n, numbers; SBP, systolic blood pressure.

**Supplementary Table 2:** Dispersion of myocardial work indices stratified by sex and age group.

|  |  | **GWI** | | | **GCW** | | | **GWW** | | | **GWE** | | |
| --- | --- | --- | --- | --- | --- | --- | --- | --- | --- | --- | --- | --- | --- |
| **Sex** | **Age group** | **Median** | **IQR** | **MAD** | **Median** | **IQR** | **MAD** | **Median** | **IQR** | **MAD** | **Median** | **IQR** | **MAD** |
| **Female** | 20–49 | 2016 | 320 | 160 | 2275 | 347 | 187 | 86 | 49 | 24 | 0.96 | 0.02 | 0.01 |
|  | 50–59 | 2017 | 403 | 206 | 2326 | 425 | 218 | 101 | 79 | 35 | 0.95 | 0.03 | 0.01 |
|  | 60–69 | 2034 | 418 | 212 | 2362 | 454 | 226 | 116 | 72 | 36 | 0.95 | 0.03 | 0.01 |
|  | 70+ | 2010 | 392 | 190 | 2372 | 374 | 181 | 134 | 104 | 47 | 0.94 | 0.04 | 0.02 |
| **Male** | 20–49 | 1929 | 434 | 219 | 2276 | 426 | 221 | 90 | 52 | 26 | 0.96 | 0.02 | 0.01 |
|  | 50–59 | 1940 | 409 | 205 | 2268 | 422 | 214 | 102 | 68 | 30 | 0.95 | 0.02 | 0.01 |
|  | 60–69 | 1853 | 377 | 189 | 2262 | 437 | 220 | 114 | 77 | 34 | 0.95 | 0.03 | 0.01 |
|  | 70+ | 1894 | 500 | 258 | 2328 | 538 | 271 | 124 | 74 | 34 | 0.94 | 0.04 | 0.02 |

Abbreviations: GCW, global constructive work; GWE, global work efficiency; GWI, global work index; GWW, global wasted work, IQR, interquartile range; MAD, mean absolute deviation.

**Supplementary Table 3.** Interobserver Reproducibility for Myocardial Work Indices.

| **MWI** | **Values per Observer** | | |  | **Agreement** | **Reliability** |
| --- | --- | --- | --- | --- | --- | --- |
|  | **HD** | **BG** | **IY** | **Pair** | **Bias (95% LOA)** | **ICC (95% CI)** |
| GWI (mmHg%) | 1963 ± 322 | 2029 ± 340 | 1949 ± 299 | HD–BG | −61 (-385–263) | 0.88 (0.82–0.92) |
|  |  |  |  | HD–IY | 18 (-234–273) |  |
|  |  |  |  | BG–IY | 80 (-210–371) |  |
| GCW (mmHg%) | 2235 (482) | 2354 (597) | 2272 (464) | HD–BG | −90 (-430–249) | 0.88 (0.82–0.92) |
|  |  |  |  | HD–IY | −29 (-303–244) |  |
|  |  |  |  | BG–IY | 62 (-257–381) |  |
| GWW (mmHg%) | 84 (63) | 89 (52) | 100 (65) | HD–BG | −1 (78–76) | 0.73 (0.62–0-81) |
|  |  |  |  | HD–IY | −26 (-125–74) |  |
|  |  |  |  | BG–IY | −24 (-125–78) |  |
| GWE (%) | 96 (2.0) | 96 (2.0) | 95 (3.0) | HD–BG | 0 (-3–3) | 0.74 (0.63–0.82) |
|  |  |  |  | HD–IY | 1 (-3–5) |  |
|  |  |  |  | BG–IY | 1 (-3–5) |  |

Myocardial work indices (MWI) are presented as mean ± SD or median (IQR), respectively. Abbreviations: BG, HD and IY, the three different observers; CI, confidence interval; GCW, global constructive work; GWE, global work efficiency; GWI, global work index; GWW, global wasted work; ICC, intraclass correlation coefficient; LOA, limits of agreement; SD, standard deviation.

**Supplementary Table 4.** Intraobserver Repeatability for Myocardial Work Indices.

| **MWI** | **Values per Observer** | | **Agreement** | **Reliability** |
| --- | --- | --- | --- | --- |
|  | **1^st^ analyses** | **2^nd^ analyses**  **(delay >4 weeks)** | **Bias (95% LOA)** | **ICC (95% CI)** |
| GWI (mmHg%) | 1984 ± 323 | 2025 ± 348 | −41 (-257–176) | 0.94 (0.87–0.97) |
| GCW (mmHg%) | 2274 (561) | 2322 (557) | −33 (-268–201) | 0.94 (0.89–0.97) |
| GWW (mmHg%) | 94 (48) | 96 (77) | 5 (-88–98) | 0.78 (0.59–0.89) |
| GWE (%) | 96 (2.0) | 96 (3.5) | 0 (-4–3) | 0.79 (0.61–0.90) |

Myocardial work indices (MWI) are presented as mean ± SD or median (IQR), respectively. Abbreviations: CI, confidence interval; GCW, global constructive work; GWE, global work efficiency; GWI, global work index; GWW, global work waste; ICC, intraclass correlation coefficient; LOA, limits of agreement; MWI, myocardial work indices; SD, standard deviation.

**Supplementary Table 5. Reproducibility of Valvular Event Timing.**

| **Valve Event** | **Correction Status** | **Intraobserver** | **Interobserver** |
| --- | --- | --- | --- |
| MVC | Before correction | -0.1 ± 7.2 | HD-BG: -0.3 ± 10 |
|  |  |  | HD-IY: -0.1 ± 8.9 |
|  |  |  | BG-IY: 0.3 ± 9.5 |
|  | After correction | -0.6 ± 6.8 | HD-BG: -1.2 ± 9.1 |
|  |  |  | HD-IY: 0.9 ± 7.3 |
|  |  |  | BG-IY: 2.2 ± 6.2 |
| AVO | Before correction | 7.1 ± 28 | HD-BG: -7.2 ± 19 |
|  |  |  | HD-IY: -11 ± 23 |
|  |  |  | BG-IY: -3.7 ± 20 |
|  | After correction | 2.9 ± 17 | HD-BG: -2.8 ± 16 |
|  |  |  | HD-IY: 1.8 ± 19 |
|  |  |  | BG-IY: 4.5 ± 12 |
| AVC | Before correction | -2.0 ± 15 | HD-BG: 1.8 ± 15 |
|  |  |  | HD-IY: -11 ± 19 |
|  |  |  | BG-IY: -13 ± 21 |
|  | After correction | -1.0 ± 14 | HD-BG: 1.2 ± 14 |
|  |  |  | HD-IY: -6.2 ± 13 |
|  |  |  | BG-IY: -7.4 ± 14 |
| MVO | Before correction | 1.8 ± 9.6 | HD-BG: -4.0 ± 14 |
|  |  |  | HD-IY: -7.2 ± 13 |
|  |  |  | BG-IY: -3.2 ± 14 |
|  | After correction | 1.8 ± 9.7 | HD-BG: -6.3 ± 11 |
|  |  |  | HD-IY: -5.0 ± 13 |
|  |  |  | BG-IY: 1.3 ± 12 |

Intra- and interobserver are presented with bias ± SD. Intraobserver reproducibility was assessed by comparing measurements from the same observer at different time points separated by >4 weeks. After correction represents comparison of observations where the same methodology for event timing was used. Abbreviations: AVC, aortic valve closure; AVO, aortic valve opening; BG, HD and IY, observers; MVC, mitral valve closure; MVO, mitral valve opening.

**Supplementary Table 6.** Predictors of Myocardial Work Indices.

| **Predictor** | **GWI (mmHg%)** | | **GCW (mmHg%)** | | **GWW (mmHg%)** | | **GWE (%)** | |
| --- | --- | --- | --- | --- | --- | --- | --- | --- |
|  | **β (95% CI)** | **p-value** | **β (95% CI)** | **p-value** | **β (95% CI)** | **p-value** | **β (95% CI)** | **p-value** |
| SBP | 15.7 (15.0–16.3) | <0.001 | 18.5 (18.0–19.1) | <0.001 | 0.011 (0.009–0.013) | <0.001 | -0.003 (-0.005–-0.001) | 0.003 |
| GLS | -67.6 (-73.0–-62.2) | <0.001 | -80.5 (-86.0–-74.9) | <0.001 | 0.040 (0.027–0.053) | <0.001 | -0.069 (-0.081–-0.057) | <0.001 |
| MVC | -0.0 (-0.8–0.8) | 0.95 | 0.7 (-0.5–1.8) | 0.27 | −0.003 (-0.006–-0.001) | 0.008 | 0.003 (0.001–0.005) | 0.003 |
| AVO | -3.7 (-4.3–-3.2) | <0.001 | −4.0 (-4.5–-3.5) | <0.001 | −0.001 (-0.003–0.000) | 0.15 | −0.001 (-0.002–0.001) | 0.48 |
| AVC | 1.2 (0.6–1.9) | <0.001 | 1.0 (0.4–1.7) | 0.002 | 0.009 (0.007–0.011) | <0.001 | −0.008 (-0.010–-0.005) | <0.001 |
| MVO | -0.9 (−1.5–-0.3) | 0.004 | 1.2 (0.6–1.8) | <0.001 | −0.001 (-0.002–0.001) | 0.52 | 0.001 (-0.001–0.003) | 0.17 |
| Age | -3.8 (−4.6–-2.9) | <0.001 | −2.6 (-3.5–-1.8) | <0.001 | 0.002 (–0.001–0.005) | 0.11 | −0.003 (-0.006–-0.001) | 0.007 |
| Sex | -130.6 (−154.2–-107.0) | <0.001 | −47.7 (-68.0–-27.3) | <0.001 | 0.087 (0.024–0.151) | 0.007 | −0.100 (-0.158–-0.041) | 0.001 |
| BMI | 1.8 (−1.0–4.5) | 0.20 | −0.1 (-2.7–2.4) | 0.91 | −0.011 (-0.019–-0.003) | 0.009 | 0.010 (0.002–0.017) | 0.01 |
| HR | -5.0 (−6.2–-3.7) | <0.001 | 1.5 (0.4–2.7) | 0.01 | 0.018 (0.015–0.022) | <0.001 | −0.016 (-0.019–-0.012) | <0.001 |
| Model fit: | 0.78 | <0.001 | 0.85 | <0.001 | 0.33 | <0.001 | 0.30 | <0.001 |

The β-coefficients are presented with corresponding p-values. GWW is log transformed and GWE is logit transformed. A negative value for sex indicates lower values in males compared to females. Abbreviations: BMI, body mass index; GCW, global constructive work; GLS, global longitudinal strain; GWE, global work efficiency; GWI, global work index; GWW, global wasted work; HR, heart rate; SBP, systolic blood pressure.

**Supplementary Table 7.** Short time delay impact of age and sex effect

| **MWI** | **Age effect, β (95% CI), p** | **Sex effect, β (95% CI), p** |
| --- | --- | --- |
| GWI | 2.4 (-0.9–5.8), 0.165 | -17 (-98–64), 0.678 |
| GCW | 2.3 (-1.2–5.7), 0.201 | -48 (-136–40), 0.282 |
| GWW (log) | -0.002 (-0.007–0.004), 0.583 | -0.15 (-0.28– -0.025), 0.019 |
| GWE (logit) | 0.002 (-0.002–0.007), 0.342 | 0.12 (0.002–0.230), 0.046 |

Short time delay was defined as ≤ 31 days between blood pressure measurements and the echocardiographic examination (n = 301). The β-coefficients are presented with corresponding p-values. Abbreviations: GCW, global constructive work; GWE, global work efficiency; GWI, global work index; GWW, global wasted work.

**Supplementary Table 8.** Summary of principal studies providing reference values for myocardial work in healthy individuals.

| **Study** | **Population (female)** | **Age (years)** | **Timing method** | **GWI (mmHg%)** | **GCW (mmHg%)** | **GWW (mmHg%)** | **GWE (%)** | **Effect of higher age** | **Sex effect** |
| --- | --- | --- | --- | --- | --- | --- | --- | --- | --- |
| Manganaro et al. (1) | 226 (62%) | 45 ± 13 | PW Doppler/ Visual | 1896 ± 308 | 2232 ± 331 | 79 (53–122) | 96 (94–97) | F: GWI and GCW higher. M: ns. | F vs. M 20-40 years: GWW higher and GWE lower. |
| Galli et al. (2) | 115 (32%) | 36 ± 13 | Visual | 1926 ± 247 | 2224 ± 229 | 90 (61–123) | 96 (94–97) | F+M: ns. | F vs. M: GWI and GCW higher. |
| Morbach et al. (3) | 779 (59%) | 49 ± 10 | CW Doppler/ Visual | 2209 (307) | 2430 (351) | 74 (54–101) | 96 (95–97) | F+M >45 years: GWW higher and GWE lower. GWI and GCW higher if >45 vs. <45 years. | F vs. M: GWI higher. |
| Olsen et al. (4) | 1827 (61%) | 45 (32-57) | TD Doppler | 2118 ± 277 | 2262 ± 283 | 64 (47–89) | 97 (96–98) | F+M: GCW higher. F: GWI and GWW (higher) and GWE (lower) with curvilinear association. | F vs. M: Higher GWI, GCW and GWW higher and GWE lower. |
| Yttervoll et al. (current study) | 1239 (55%) | 57 ± 12 | Doppler/ Visual | 1975 ± 310 | 2318 ± 334 | 104 (73–146) | 95 (94–96) | F+M: GCW and GWW higher.  GWI and GWE lower. (F: GWI ns. M: GCW ns.) | F vs. M: GWI and GCW higher. |

Populations are presented as N total (% women), others are presented as mean ± SD or median (IQR). Abbreviations: CW, continuous wave; F, female; F+M, female and male analyzed together; GCW, global constructive work; GWE, global work efficiency; GWI, global work index; GWW, global wasted work; M, male; ns, not significant; PW, pulsed wave, TD, tissue Doppler.

# **REFERENCES**

1. Manganaro R, Marchetta S, Dulgheru R, Ilardi F, Sugimoto T, Robinet S, et al. Echocardiographic reference ranges for normal non-invasive myocardial work indices: Results from the EACVI NORRE study. European Heart Journal Cardiovascular Imaging. 2019;20(5).

2. Galli E, John-Matthwes B, Rousseau C, Schnell F, Leclercq C, Donal E. Echocardiographic reference ranges for myocardial work in healthy subjects: A preliminary study. Echocardiography. 2019;36(10).

3. Morbach C, Sahiti F, Tiffe T, Cejka V, Eichner FA, Gelbrich G, et al. Myocardial work - correlation patterns and reference values from the population-based STAAB cohort study. PLoS ONE. 2020;15(10 October).

4. Olsen FJ, Skaarup KG, Højbjerg Lassen MC, Johansen ND, Sengeløv M, Jensen GB, et al. Normal Values for Myocardial Work Indices Derived From Pressure-Strain Loop Analyses: From the CCHS. Circulation: Cardiovascular Imaging. 2022;15(5).
